# Enhanced CO_2_ Separation Performance of Mixed Matrix Membranes with Pebax and Amino-Functionalized Carbon Nitride Nanosheets

**DOI:** 10.3390/membranes15100306

**Published:** 2025-10-07

**Authors:** Mengran Hua, Qinqin Sun, Na Li, Mingchao Zhu, Yongze Lu, Zhaoxia Hu, Shouwen Chen

**Affiliations:** 1 Jiangsu Key Laboratory of Chemical Pollution Control and Resources Reuse, School of Environmental and Biological Engineering, Nanjing University of Science and Technology, Nanjing 210094, China; h19855590299@163.com (M.H.); 15051990131@163.com (Q.S.); mczhu@xzmu.edu.cn (M.Z.); huzhaoxia@njust.edu.cn (Z.H.); chensw@njust.edu.cn (S.C.); 2 School of Energy and Environment, Southeast University, Nanjing 210096, China; 3 Key Laboratory of Water Pollution Control and Ecological Restoration of Xizang, National Ethnic Affairs Commission, Xizang Minzu University, Xianyang 712082, China; 4 Information Engineer College, Xizang Minzu University, Xianyang 712082, China

**Keywords:** g-C_3_N_4_ nanosheets, mixed matrix membrane, gas separation, amino-functionalized, selectivity

## Abstract

Highly permeable and selective membranes are crucial for energy-efficient gas separation. Two-dimensional (2D) graphitic carbon nitride (g-C_3_N_4_) has attracted significant attention due to its unique structural characteristics, including ultra-thin thickness, inherent surface porosity, and abundant amine groups. However, the interfacial defects caused by poor compatibility between g-C_3_N_4_ and polymers deteriorate the separation performance of membrane materials. In this study, amino-functionalized g-C_3_N_4_ nanosheets (CN@PEI) was prepared by a post-synthesis method, then blended with the polymer Pebax to fabricate Pebax/CN@PEI mixed matrix membranes (MMMs). Compared to g-C_3_N_4_, MMMs with CN@PEI loading of 20 wt% as nanofiller exhibited a CO_2_ permeance of 241 Barrer as well as the CO_2_/CH_4_ and CO_2_/N_2_ selectivity of 39.7 and 61.2, respectively, at the feed gas pressure of 2 bar, which approaches the 2008 Robeson upper bound and exceeded the 1991 Robeson upper bound. The Pebax/CN@PEI (20) membrane showed robust stability performance over 70 h continuous gas permeability testing, and no significant decline was observed. SEM characterization revealed a uniform dispersion of CN@PEI throughout the Pebax matrix, demonstrating excellent interfacial compatibility between the components. The increased free volume fraction, enhanced solubility, and higher diffusion coefficient demonstrated that the incorporation of CN@PEI nanosheets introduced more CO_2_-philic amino groups and disrupted the chain packing of the Pebax matrix, thereby creating additional diffusion channels and facilitating CO_2_ transport.

## 1. Introduction

Statistical data indicate that CO_2_ is the principal contributor to the greenhouse effect, accounting for more than 70% of global greenhouse gas emissions, while methane, nitrous oxide, and various fluorinated gases collectively comprise the remainder [1]. The climate change resulting from global warming and excessive CO_2_ emissions has attracted considerable attention towards CO_2_ capture [2]. In response to these environmental challenges, China has developed a strategic plan targeting carbon peaking and carbon neutrality, with a specific emphasis on reducing CO_2_ emissions. In recent years, membrane technology for gas separation has garnered considerable attention due to its processing versatility, energy efficiency, low capital and operational cost, and compact footprint, making it a promising CO_2_ separation technology [3,4]. Contemporary separation membranes predominantly comprise inorganic membranes, polymeric membranes, and mixed matrix membranes (MMMs) [5,6,7]. Among these, MMMs have attracted widespread attention for gas separation applications owing to their combination of the dimensional stability and efficient gas separation capabilities inherent to inorganic materials, alongside the ease of fabrication and low cost of polymer materials [8,9]. In the past few decades, extensive research efforts have been devoted to investigating diverse inorganic materials for the fabrication of MMMs, which exhibit superior gas separation performance compared to conventional polymeric and inorganic membranes. Numerous inorganic materials show significant potential as fillers for membrane applications [10]. Among these, two-dimensional (2D) materials have emerged as particularly promising, driving notable advancements in membrane design. These materials have garnered considerable attention due to their distinctive physicochemical structures, including sub-nanometer thicknesses, high aspect ratios, and diverse surface chemistries [11,12]. A variety of 2D materials, such as zeolites [13], metal–organic frameworks (MOFs) [14], covalent organic frameworks (COFs) [15], MXenes [16], and graphitic carbon nitride (g-C_3_N_4_) [17], have shown unprecedented potential in applications of CO_2_/CH_4_ and CO_2_/N_2_ separation, with permeability values ranging from 400 to 10,528 Barrer and selectivity values ranging from 41 to 47.8 [18].

Notably, g-C_3_N_4_, with its abundant gas transport channels, excellent mechanical strength, and thermal stability, has been considered as a promising candidate for separation membranes [19]. g-C_3_N_4_ possesses a planar, two-dimensional layered structure analogous to that of graphite, where adjacent atomic layers are interconnected by van der Waals forces [20]. Furthermore, g-C_3_N_4_ comprises intrinsic pores formed by triazine units or tri-s-triazine units (geometric diameter of 3.11 Å), alongside structurally defective pores (pore diameters of 3.1–3.4 Å) formed during thermal polymerization, which provide additional nano-transportation channels and sieving properties [18,21]. In addition, g-C_3_N_4_ nanosheets possess abundant amine groups that exhibit a strong affinity for CO_2_ [22], which enhances selective transport and then facilitates efficient CO_2_ separation. Tian et al. [23] prepared a variety of mixed matrix membranes (MMMs) by incorporating g-C_3_N_4_ nanosheets into the matrix of polymers of intrinsic microporosity (PIM-1). Compared to the pure PIM-1 membrane, the periodic ultramicropores of g-C_3_N_4_ exhibited a size-sieving effect, preferentially facilitating the transport of smaller molecules (such as H_2_). Moreover, the selectivity for H_2_/CH_4_ and H_2_/N_2_ were both enhanced without compromising gas permeability. Niu et al. [24] constructed a novel type of supported ionic liquid membrane (SILM) for CO_2_/N_2_ and CO_2_/CH_4_ separation by nanoconfining 1-ethyl-3-methylimidazole acetate ([EMIm][AcO], IL) within the two-dimentional (2D) channels of the g-C_3_N_4_ laminated membrane. The results indicated that g-C_3_N_4_ SILM achieved a maximum CO_2_ permeance of 992.97 GPU for CO_2_/N_2_ separation and 1160.52 GPU for CO_2_/CH_4_ separation under 1 bar and 25 °C. Meanwhile, the g-C_3_N_4_ SILM demonstrated excellent selectivity for CO_2_/N_2_ (52.49) and CO_2_/CH_4_ (48.41). Despite these advantages of g-C_3_N_4_, the material’s inherent ultramicropores (<3.4 Å) preferentially facilitate smaller gas molecules (e.g., H_2_), while presenting significant challenges in fabricating large areas and retaining the structural integrity of its two-dimensional plane during the delamination of bulky g-C_3_N_4_ into nanosheets. Additionally, challenges including poor dispersion of nanosheets, weak interfacial compatibility with polymer matrices, and interlayer defects within stacked nanosheet architectures must be addressed [25]. Therefore, surface modification of g-C_3_N_4_ nanosheets becomes imperative to improve interfacial compatibility with organic polymers and optimize CO_2_-selective transport pathways through the MMMs.

In recent years, amino-functionalized fillers have been widely used as materials for CO_2_ capture and selective separation. Among these, polyethyleneimine (PEI), characterized as a long-branched polymer, is noteworthy for its high density of amine groups and readily accessible primary amino groups along its chain [26]. Owing to its highly branched structure featuring abundant -NH_2_ groups and terminal primary amine sites, PEI is widely employed as a cross-linker for both heavy metals removal and CO_2_ adsorption [27]. These structural properties make PEI an ideal polyamine compound for grafting onto g-C_3_N_4_ [28]. Moreover, PEI modification significantly enhances the compatibility between g-C_3_N_4_ nanosheets and the Pebax matrix, resulting in superior dispersion of the nanofiller throughout the polymer composite. Jiao et al. [29] synthesized well-dispersed PEI-functionalized ZIF-8 nanoparticles (PEI-ZIF-8, with a particle size of about 15 nm) through an in situ synthesis approach, which were subsequently incorporated as nanofillers to fabricate Pebax/PEI-ZIF-8 composite membranes. Results demonstrated that the Pebax/PEI-ZIF-8 composite membrane exhibited a CO_2_ permeance of 13.0 GPU with a CO_2_/N_2_ selectivity of 49.0, corresponding to significant enhancements of 80.6% and 118.8% in selectivity compared to the unmodified Pebax membrane. Wang et al. [30] effectively enhanced the compatibility between multi-walled carbon nanotubes (MWCNTs) and Pebax-1657 using a PEI surface functionalization strategy. Correspondingly, the prepared PEI@MWCNTs/Pebax-1657 MMMs showed 178.08% and 152.49% enhancement in CO_2_/N_2_ selectivity and permeability of CO_2_ compared to the unmodified membrane. While several studies have investigated the interactions of PEI with various materials [29,30], the interfacial interactions between PEI and g-C_3_N_4_ remain poorly understood. More importantly, their specific effects on the CO_2_/CH_4_ separation performance of MMMs remain unexplored and warrant in-depth study.

The objective of this study was to (1) synthesize amino-functionalized g-C_3_N_4_ nanosheets through polyethyleneimine (PEI) modification, and (2) incorporate these nanostructures as functional fillers into a Pebax matrix to fabricate high-performance mixed matrix membranes (MMMs). Specifically, CN@PEI was synthesized by employing post-synthesis modification to incorporate amino groups into g-C_3_N_4_ nanosheets. The mixed matrix membranes (MMMs) were fabricated via solution casting, incorporating the PEI-functionalized g-C_3_N_4_ nanosheets as nanofillers within the polymer matrix. The fabricated MMM series were systematically characterized using XRD, FTIR, SEM, and TGA to evaluate their structural and thermal properties. Then, the impacts of PEI surface modification and nanofiller loading content on the membrane characteristics including interfacial morphology, mechanical strength, and thermal stability, were comprehensively investigated. The permeability and selectivity of the membranes towards CO_2_ were also explored. These results might bridge an important knowledge gap in g-C_3_N_4_ nanosheet applications for gas separation, while providing fundamental insights for designing novel separation membranes.

## 2. Materials and Methods

### 2.1. Materials

Melamine (99%), ammonium persulfate ((NH_4_)_2_S_2_O_8_, AR), and ethanol (EtOH, AR) were purchased from Middle East Chemical Glass Instrument Co., Ltd. in Nanjing, Jiangsu Province, China. Ammonium chloride (NH_4_Cl, AR), zinc chloride (ZnCl_2_, 98%), dimethyl sulfoxide (DMSO), hydrochloric acid (HCl, 36%~38%), and sodium hydroxide (NaOH, AR) were obtained from Sinopharm Chemical reagent Co., Ltd. in Shanghai, China. 1, 4-Butane sultone (AR) was supplied by Dibao Biotechnology Co., Ltd. in Shanghai, China. Poly(ether-block-amide) (Pebax-1657) was purchased from The French company Arkema. Maleic anhydride (C_4_H_2_O_3_, 99%) was obtained from Juyou Scientific Equipment Co., Ltd. in Nanjing, Jiangsu Province, China. Polyethyleneimine (PEI, 30%) was supplied by Lattice Chemical Technology Co., Ltd. in Nanjing, Jiangsu Province, China. Methanol (CH_3_OH, AR) was purchased from Wanqing glass instrument Co., Ltd. in Nanjing, Jiangsu Province, China. Deionized (DI) water was used for all experimental procedures.

### 2.2. Preparation of g-C_3_N_4_ (CN) Nanosheets

The CN nanosheets were synthesized through thermal polymerization. Specifically, 2.0 g of melamine and 4.0 g of NH_4_Cl were mixed and ground together before being placed into a ceramic crucible. The mixture was then heated in a muffle furnace at 550 °C for 4 h, with both a heating and cooling rate of 5 °C/min. The resulting bulk g-C_3_N_4_ (light yellow product) was milled and finely ground into powder for further use.

### 2.3. Preparation of Amino-Functionalized CN Nanosheets

Firstly, 0.5 g of pristine CN nanosheet powder was dispersed in 20 mL of DMSO solution, followed by the addition of 0.5 g of maleic anhydride and 0.1 g of ammonium persulfate. The mixture was transferred to a three-neck flask, heated to 80 °C under an N_2_ atmosphere, and stirred for 2 h. Afterward, 0.4 g of PEI solution was added by pipetting while maintaining the temperature at 80 °C, and the reaction was allowed to proceed for an additional 4 h. Upon completion, the resultant brown powder was collected by centrifugation, washed three times with methanol and DI, respectively, and then dried in a vacuum at 100 °C for 24 h. The final product obtained was designated as CN@PEI.

### 2.4. Preparation of the Membranes

**Figure 1:**
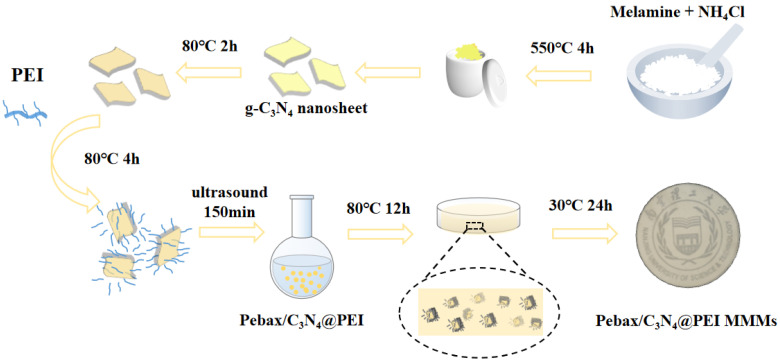
Schematic diagram of the preparation process and gas transport mechanism of Pebax/CN@PEI MMMs.

The MMMs of varying loadings were prepared using the solution casting method, as illustrated in Figure 1. Initially, a quantity of Pebax microspheres were first taken and added to a mixture of ethanol and water (volume ratio 7:3). The microspheres were then dissolved by refluxing for 2 h at 80 °C to obtain a Pebax solution with a concentration of 5 wt%. Subsequently, a certain amount of CN@PEI was dispersed and treated with ultrasonication for 30 min in the same mixed solvent of ethanol and water, which was repeated 5 times to obtain a uniformly dispersed filler solution. The resulting solutions were separately combined with the Pebax solution and stirred at reflux for 12 h at 80 °C to produce the casting solution. After degassing by ultrasound treatment, the casting solutions were poured into a glass membrane module and dried under vacuum at 30 °C for 24 h. The mass percentage of filler in the membrane was varied at 0 wt%, 10 wt%, 15 wt%, 20 wt%, and 25 wt% to fabricate membranes with different loadings. For comparison, pure Pebax membranes were fabricated using the same manufacturing method and drying process by the aforementioned procedure. The thickness of the MMMs ranged between 35 and 45 μm. The MMMs were denoted as Pebax/CN@PEI(x), where x (=10, 15, 20, 25) represented the content (wt%) of nanofillers in the Pebax polymer matrix.

### 2.5. Characterization of Nanofillers and MMMs

The micromorphology of the nanofillers and synthetic membranes was analyzed using a field emission scanning electron microscope (SEM, FEI Quanta 250F, FEI Company, USA). The crystal structure of the nanofillers was examined by X-ray diffractometer (XRD, Bruker-AXS D8 Advance, Karlsruhe, Germany) with measurement angles ranging from 5° to 80° and a scanning rate of 5°∙min^−1^. Infrared spectroscopy tests were conducted using a Fourier transform infrared spectrometer (FTIR, Bruker Vector 22, Bremen, Germany), scanning a wavelength range of 4000 to 500 cm^−1^. The pore structure, gas adsorption, and desorption processes of the nanofillers were characterized by a Brunauer–Emmett–Teller (BET) surface area and pore size distribution analyzer (BSD-PM, Northstone, Frankfurt, Germany). The thermal stability of the membranes was analyzed using a thermogravimetric analysis (TGA) instrument (Hitachi 7300, Hitachi Limited, Tokyo, Japan), with a temperature range of 30–700 °C and a heating rate of 10 °C min^−1^ under an N_2_ atmosphere. The glass transition temperature (*T_g_*) of the membranes was examined by a differential scanning calorimeter (DSC, Q2000, TA Instruments, New Castle, Delaware, USA) over a temperature range of −80 °C to 200 °C, maintaining a temperature increase rate of 10 °C/min and an N_2_ atmosphere. The mechanical properties of the membranes were tested using a universal material tester (GS-X 34905489A, Shimadzu Corporation, Kyoto, Japan). The composition of the gases permeating through the mixed matrix membrane (MMM) was analyzed using gas chromatography (Agilent 7890A), equipped with a thermal conductivity detector (TCD), using nitrogen at a flow rate of 50 mL/min as the carrier gas, with the column temperature set at 100 °C, the injector temperature at 150 °C, and the detector temperature at 250 °C. The densities of the nanofillers and membranes were determined by specific gravity bottles. The fractional free volume (FFV) was characterized using density data and applying the following equation [31,32]:

(1) $$FFV=\frac{V-V_{0}}{V}=1-\rho V_{0}$$

where V represents the specific volume of the polymer or mixed system (cm^3^/g), while $V_{0}$ corresponds to the volume occupied by the polymer molecules (cm^3^/g) at 0 K. The calculation of $V_{0}$ involves using the van der Waals volume ($V_{w}$) and applying the Bondi group contribution approach:

(2) $$V_{0}=1.3V_{w}$$

According to the calculation, the $V_{w}$ value of Pebax MH1657, which consists of 40% polyamide (PA) and 60% polyether (PE) segment, was 0.546 cm^3^/g [33]. The FFV of the filler (${FFV}_{filler}$) is determined by multiplying the pore volume (cm^3^/g) as measured by the BET adsorption analysis, by the particle density (g/cm^3^) [28]. The FFV of the MMMs is then simply calculated by the following equation [33]:

(3) $${FFV}_{MMMs}={FFV}_{polymer}\left(\phi_{polymer}\right)+{FFV}_{filler}\left(\phi_{filler}\right)$$

where ϕ represents the volume fraction of polymer and filler in the membrane, which is determined from the density and their mass fraction (0, 10, 15, 20, and 25 wt%).

### 2.6. Gas Permeation Experiments

**Figure 2:**
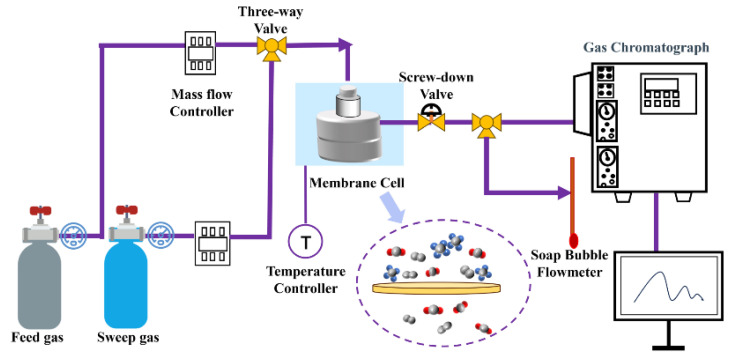
Schematic diagram of mixed gas permeation setup.

The schematic diagram of the gas separation device is presented in Figure 2. Three pure gas species (N_2_, CH_4_, and CO_2_) were applied as the test gases. All tests were conducted using a custom-made permeation cell, which possessed an effective membrane area of 6.69 cm^2^. The feed gas could be either a mixed gas or pure gas. In Figure 2, the purple arrow indicates the permeation process of different gases through the gas separation membrane. To assess the membrane’s performance in gas separation, a CO_2_/CH_4_ mixture with a volume ratio of 50/50% was used as the feed gas, while N_2_ (50 mL/min) served as the sweep gas on the downstream side. All pure and mixed gas permeation tests were conducted at ambient temperature (25 °C) with a trans-membrane pressure control of 0.2 Mpa. Steady-state feed conditions were ensured during the tests, and the composition of the permeate gases was determined using gas chromatography (GC, Agilent 3420A, Agilent Technologies Inc., Santa Clara, CA, USA). The pure gas and mixed gas permeation data for each membrane were collected from 3 distinct samples of the membrane, synthesized exactly using identical procedures. Each test was repeated 4–6 times under the same conditions, and the averages and standard deviations were reported. The gas permeability and selectivity coefficient were used to evaluate the gas separation performance of the mix matrix membranes prepared in this experiment.

The gas permeability (P) was calculated by Equation (4) [34]:

(4) $$P_{i}=\frac{LQ_{i}}{∆P_{i}A}$$

where L is the thickness of the membrane (cm), $Q_{i}$ is the volumetric flow rate of gas component i (cm^3^(STP) s^−1^), $∆P_{i}$ is the differential partial pressure of gas i across the membrane (cmHg), A is the effective membrane area (cm^2^), and the unit of the permeability coefficient ${(P}_{i})$ is generally denoted as Barrer (1 Barrer = 10^−10^ cm^3^ (STP) cm/(cm^2^ s cmHg)).

The ideal selectivity ($\alpha_{i/j}$) for a pair of gases is defined as their permeability ratio [35]:

(5) $$\alpha_{i/j}=\frac{P_{i}}{P_{j}}$$

where $P_{i}$ and $P_{j}$ represent the permeability of individual gas components i and j, respectively.

The selectivity of a binary gas mixture can be calculated by the following equation [36]:

(6) $$\alpha_{ij}=\frac{y_{i}/y_{j}}{x_{i}/x_{j}}$$

The variables x and y denote the volume fractions of the different components i and j on the feed side and permeate sides, respectively; in this paper, $x_{i}$ = $x_{j}$ = 0.5 and y was obtained by gas chromatography analysis.

## 3. Results and Discussion

### 3.1. Characterization of the Nanofillers

**Figure 3:**
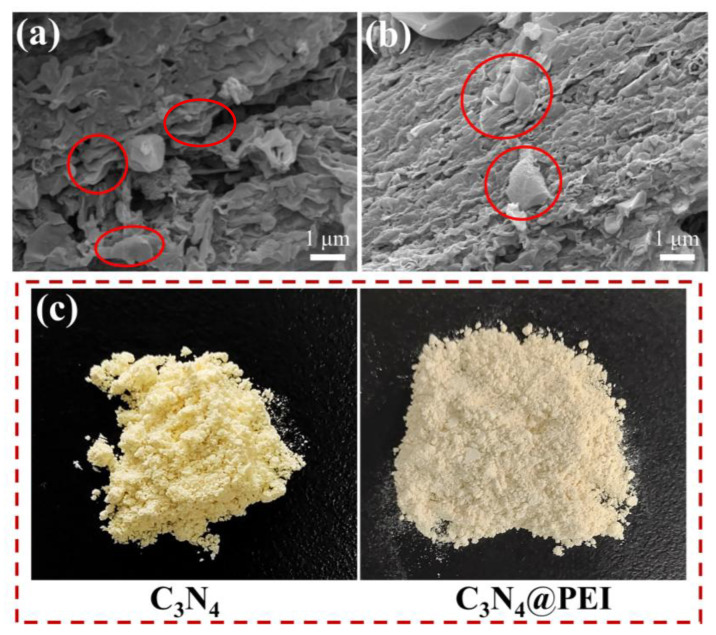
(**a**,**b**) SEM and (**c**) optical images of CN and CN@PEI nanosheets.

The structure and morphology of the prepared materials were investigated using SEM and optical photographs, as illustrated in Figure 3. The SEM images of bulk g-C_3_N_4_ depict that g-C_3_N_4_ was composed of graphite flakes, while the synthesized CN nanosheets displayed an irregular layered structure resulting from the aggregation of graphite-like planes (indicated by the red circles in Figure 3a). After modification, the nanosheets showed minimal structural changes (indicated by the red circles in Figure 3b). The structural differences between them will be elaborated in detail in the subsequent XRD images (Figure 5a). Furthermore, the modified CN@PEI nanosheets appeared darker than the control unmodified CN nanosheets (Figure 3c).

**Figure 4:**
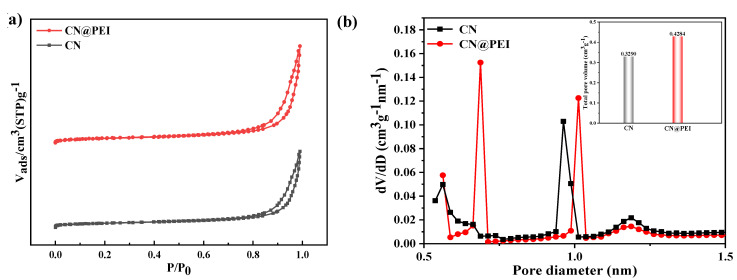
CN and CN@PEI: (**a**) N_2_ adsorption isotherms at 77 K, and (**b**) pore size distribution and total pore volume (inset).

Figure 4 depicts the N_2_ adsorption and desorption isotherms of CN and CN@PEI, as well as the pore size distribution. As Figure 4a shows, the N_2_ adsorption/desorption behavior of CN@PEI closely matched that of the pristine CN nanosheets. The specific surface area of CN@PEI nanosheets was 52.15 m^2^/g, representing a 13.5% enhancement relative to pristine CN nanosheets (45.93 m^2^/g). These results demonstrated that the surface functionalization with PEI led to an expansion in the surface area of CN nanosheets. Moreover, the pore size distribution and total pore volume of both fillers were analyzed, as shown in Figure 4b. It is evident that the pore size increased after modification, accompanied by the formation of new nanopores at 0.7 nm. Concurrently, the total pore volume increased from 0.3290 cm^3^g^−1^ (CN) to 0.4284 cm^3^g^−1^ (CN@PEI), yielding a 30.2% enhancement (inset of Figure 4b). The formation or expansion of pores might result from interactions between amino groups and functional groups on the surface of CN nanoparticles. These results demonstrated that the PEI modification of CN nanosheets significantly alters their micropore structure, forming additional adsorption sites in the CN@PEI nanosheets.

**Figure 5:**
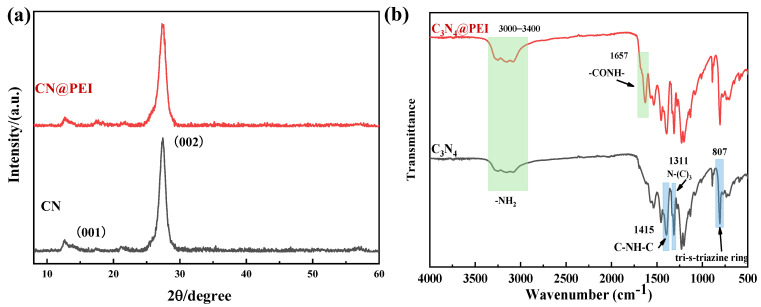
The XRD spectra (**a**) and FTIR spectra (**b**) of CN and CN@PEI.

Figure 5 displays the XRD and FTIR spectras of CN and CN@PEI nanosheets. Two distinctive diffraction peaks of CN are evident in the XRD pattern (Figure 5a). The sharp reflection peak at 27.56° corresponds to the stacking of aromatic planes (002) with a d-spacing of 0.322 nm [25]. Another peak at 12.62° with a d-spacing of 0.653 nm corresponds to the periodic repetition of tri-s-triazine units [37]. This observation further confirmed the presence of triangular nanopores in CN. Additionally, the characteristic peaks of the pristine CN nanosheets were also observed in the XRD pattern of CN@PEI nanosheets, which indicated that the structure and morphology of the pristine CN nanosheets remained largely unchanged after amino functionalization.

The chemical structure of CN nanoparticles was analyzed using FTIR. As Figure 5b shows, the FTIR spectrum of CN exhibited a prominent peak at 807 cm^−1^, corresponding to the vibration of the tri-s-triazine ring system [23]. Furthermore, vibration peaks of N-(C)_3_ and C-NH-C of g-C_3_N_4_ nanosheets were observed at 1311 cm^−1^ and 1415 cm^−1^, which were consistent with previous studies [38]. Conversely, the FTIR spectra of the modified CN@PEI nanosheets exhibited a new peak at 1657 cm^−1^, attributable to the stretching vibration of -CO-NH-, confirming the reaction between the added maleic anhydride and both CN nanosheets and PEI [39]. Moreover, the broad shoulder band in the range of 3000–3400 cm^−1^ was attributed to the -NH_2_ groups, and the amino peak of the modified CN@PEI nanosheets exhibited a significant enhancement. The characteristic peaks of CN and CN@PEI have been marked in blue and green respectively in Figure 5b. These spectral results strongly confirmed the successful grafting of PEI onto the CN nanosheets.

**Figure 6:**
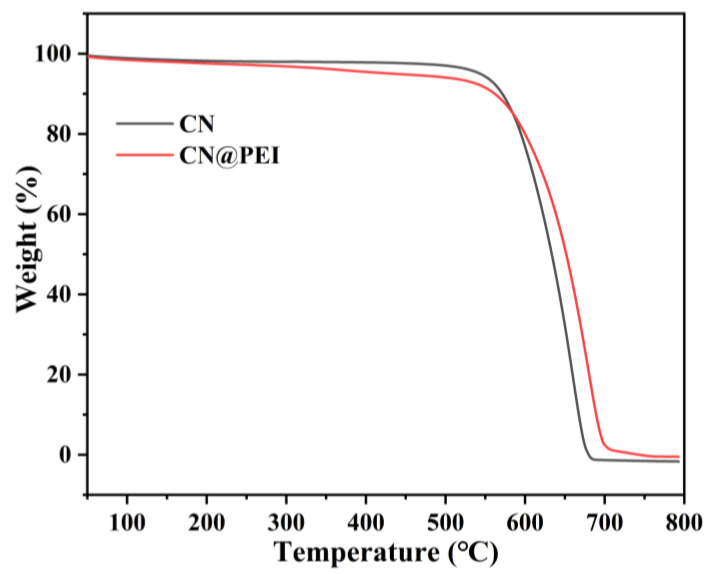
TGA curves of CN and CN@PEI nanosheets.

Figure 6 displays the TGA curves of both nanofillers, revealing their thermal stability. Both nanofillers exhibited comparable pyrolysis behavior, characterized by two distinct stages: (1) elimination of residual water and solvent (50–500 °C), and (2) the thermal decomposition of the organic skeleton of the nanofiller (500–700 °C). The slight difference in residual weight might be predominantly attributed to the stronger interactions between PEI and the CN surface, thereby stabilizing the chemical structure and improving the thermal stability of CN@PEI nanofillers, leading to improved thermal resistance during the TGA analysis.

### 3.2. Characterization of the MMMs

**Figure 7:**
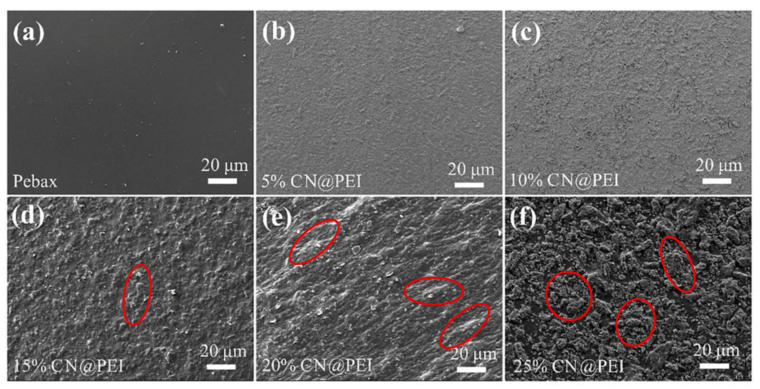
Surface SEM image of (**a**) pure Pebax membrane, and surface SEM images of Pebax/CN@PEI MMMs with loading capacity of different fillers (**b**) 5 wt%, (**c**) 10 wt%, (**d**) 15 wt%, (**e**) 20 wt%, and (**f**) 25 wt%.

**Figure 8:**
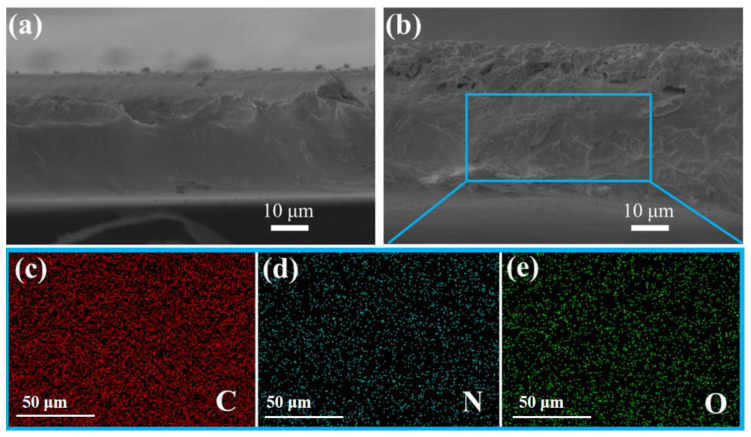
Cross-sectional SEM maps of (**a**) pure Pebax membrane; (**b**) Pebax/CN@PEI (25) membrane; and (**c**–**e**) EDS mapping images of C, O, and N elements recorded from (**b**).

MMMs with different filler loadings of CN and CN@PEI were prepared using the solution casting technique, and their morphological features are displayed in Figure 7 and Figure 8. SEM characterization was employed to analyze the interfacial morphology of both pure Pebax and Pebax/CN@PEI MMMs with different CN@PEI loadings to evaluate the nanofiller dispersion and matrix compatibility (Figure 7). As Figure 7a shows, the pure Pebax membrane exhibited a smooth surface without any voids. As the composite filler loading increased from 5 wt% to 25 wt%, the surface of the membrane became progressively rougher. Upon surpassing a loading capacity of 15 wt%, slight agglomeration of the nanofillers occurred on the membrane surface (indicated by the red circles in Figure 7d–f), particularly in the sample with 20 wt% and 25 wt% CN@PEI, while no discernible interfacial voids or defects were observed, which indicated a degree of interfacial compatibility between CN@PEI and Pebax. Moreover, the cross-sectional morphology of Pebax and Pebax/CN@PEI MMMs was also characterized by SEM (Figure 8). As Figure 8 shows, the Pebax membranes exhibited a dense structure (Figure 8a), while the Pebax/CN@PEI (25) MMMs displayed a rougher cross-sectional morphology (Figure 8b). The nanoparticles were tightly integrated with Pebax polymer matrix, and no significant voids or defects were observed. Moreover, the EDS images of the Pebax/CN@PEI (25) MMM depicts the uniform distribution of C, N, and O elements in the membranes (Figure 8c–e), further verifying the uniform dispersion of CN@PEI nanofillers within the membranes and providing additional evidence of the successful modification of CN nanosheets.

**Figure 9:**
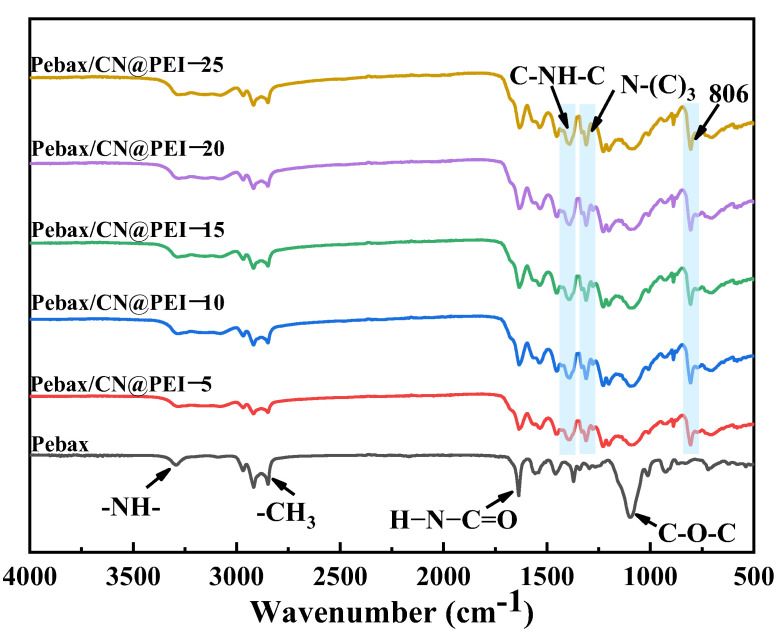
FTIR spectrogram of mixed matrix membranes.

Figure 9 shows the FTIR spectra of the pure Pebax membrane and the MMMs loaded with CN@PEI nanosheets. For the pure Pebax membrane, the vibrational peak at 1097 cm^−1^ was attributed to the tensile vibration of the C-O-C group induced by the soft segments of PEO in the Pebax phase, while the characteristic absorption peaks of the -NH- and H-N-C=O groups of the PA hard segment in the Pebax matrix appeared at 1636 and 3292 cm^−1^ [40,41]. The FTIR spectrum of CN exhibited a strong peak at 806 cm^−1^ corresponding to the vibration of the tri-s-triazine ring [42], thereby confirming the presence of the CN structure within the membrane matrix. Furthermore, distinct sharp absorption bands were also observed at 1311 cm^−1^ and 1415 cm^−1^, corresponding to the stretching vibration modes of N-(C)_3_ and C-NH-C units [23]. These results unequivocally confirmed the successful incorporation of CN@PEI nanosheets into the polymer matrix.

**Figure 10:**
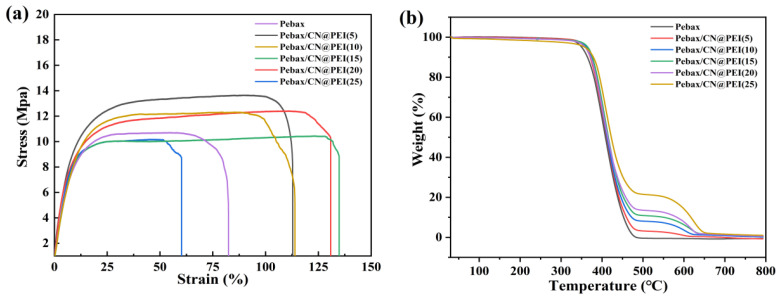
(**a**) Tensile testing and (**b**) TGA curves of pure Pebax membrane and Pebax/CN@PEI MMMs.

The stress–strain curves were employed to assess the mechanical stability of the membranes (Figure 10a and Table 1). As Table 1 shows, the addition of CN@PEI nanosheets significantly enhanced the mechanical properties of Pebax membranes, resulting in higher ultimate tensile strength and Young’s modulus. For example, compared to pure Pebax membranes, the incorporation of 5 wt% of CN@PEI nanosheets increased the tensile strength by 23.9%. Moreover, it reached its maximum value of 16.3 MPa at a content of 15 wt%, representing a 43.0% increase over that of the pure Pebax membranes (11.4 MPa). This enhancement in mechanical properties originates from strong interfacial interactions between the CN@PEI and Pebax, which facilitate efficient transfer of mechanical load to the rigid CN@PEI nanosheets. The data revealed that CN@PEI nanosheets and Pebax matrix exhibit excellent compatibility under certain loadings, allowing for effective load transfer between the continuous and dispersed phases, thereby enhancing the mechanical properties of the membranes with an appropriately controlled filler concentration. However, when CN@PEI loading increased to 25 wt%, the tensile strength and elongation at break sharply decreased, while the Young’s modulus increased. This result might be attributed to nanoparticle agglomeration at higher loadings, which resulted in chain breakage during preparation, leading to a decrease in tensile strength and elongation at break. However, strong interfacial interactions between the CN@PEI and Pebax contributed to increased Young’s modulus.

Figure 10b depicts the TGA curves of Pebax membranes and Pebax/CN@PEI mixed matrix membranes (MMMs). Both pure Pebax membranes and Pebax/CN@PEI MMMs exhibited two pyrolysis stages. The initial weight loss below 240 °C stemmed from dehydration (i.e., evaporation of free and bound water) and the volatilization of residual solvents. The degradation of the Pebax polymer backbone occurred between 400 and 500 °C, representing the primary phase of overall mass loss. Moreover, the incorporation of CN@PEI nanosheets induced a third thermal degradation stage of MMMs above 500 °C, primarily caused by the decomposition of these nanosheets. These findings underscored the enhanced thermal stability of MMMs, which resulted from the exceptional intrinsic thermal properties of CN@PEI nanosheets.

**Figure 11:**
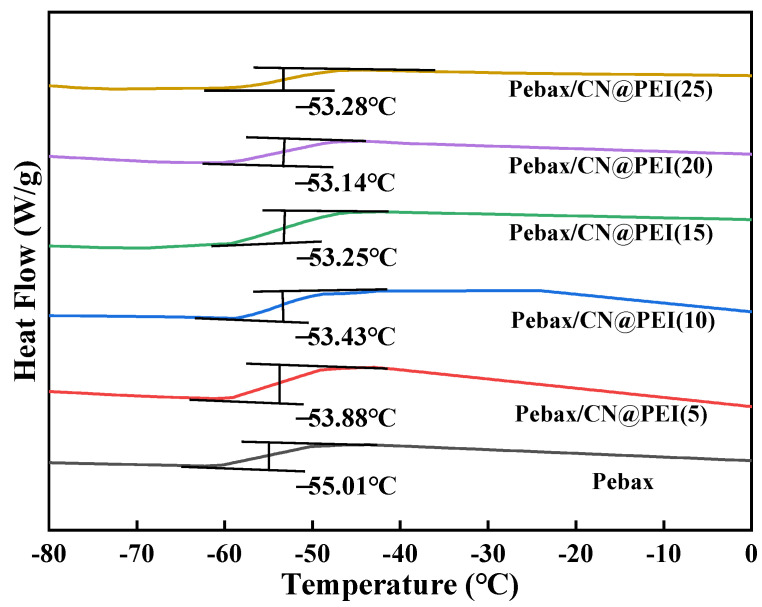
DSC curves of pure Pebax membrane and MMMs.

Differential scanning calorimetry (DSC) analysis was performed to characterize the thermal properties of both pure Pebax membranes and Pebax/CN@PEI MMMs (Figure 11 and Table S1). As the content of CN@PEI nanosheets increased from 5 wt% to 20 wt%, the *T_g_* displayed a rising trend from −53.88 °C to −53.14 °C, while the *T_g_* of the pure Pebax membrane was measured at −55.01 °C. When CN@PEI loading increased to 25 wt%, the agglomeration of the nanosheets led to the formation of interfacial defects with the polymer matrix, inducing a reduction in *T_g_*. Consequently, the elevated *T_g_* implied that the presence of CN@PEI nanosheets reduced the polymer chain flexibility. This restricted mobility of the polymer chains enhanced resistance to larger gas molecule transport, thereby improving CO_2_/CH_4_ selectivity.

The FFV of a membrane was considered one of the most critical structural parameters affecting gas transport properties [23]. Based on the measured density data, the FFV values for the prepared membranes was calculate and presented in Table 2. As Table 2 shows, the nanofiller content significantly affected the density and FFV of MMMs. Specifically, in comparison to the density of the pure Pebax membrane (1.042 g/cm^3^), the density of the membrane decreased from 1.040 g/cm^3^ to 1.034 g/cm^3^ when the CN@PEI nanofiller content increased from 5 wt% to 25 wt%. This trend could be explained by the lower density of the nanoparticles relative to the Pebax polymer and their successful incorporation into the polymer matrix, which promoted the formation of a dense layer at the polymer–filler interface known as interfacial stiffness [43]. Moreover, the introduction of fillers enlarged the free volume of the membranes, and this expansion correlated positively with increasing filler content. As the CN@PEI content increased from 5 wt% to 25 wt%, the Pebax/CN@PEI MMMs achieved a higher FFV compared to that of the pure Pebax membranes (0.260). Notably, the highest FFV reached 0.283 at CN@PEI loading of 25 wt%, representing an 8.67% increase over the pure Pebax. The incorporation of CN nanosheets with a high aspect ratio impeded the effective stacking of polymer chains and amplified the interfacial volume, resulting in higher FFV in the MMMs. Moreover, the introduction of micropores increased membrane free volume, attributable to the porous characteristics of the incorporated fillers. These micropores also provided additional pathways for gas molecules traversing, thereby enhancing gas diffusivity by providing alternative routes for gas transport.

### 3.3. Gas Separation Performance

**Figure 12:**
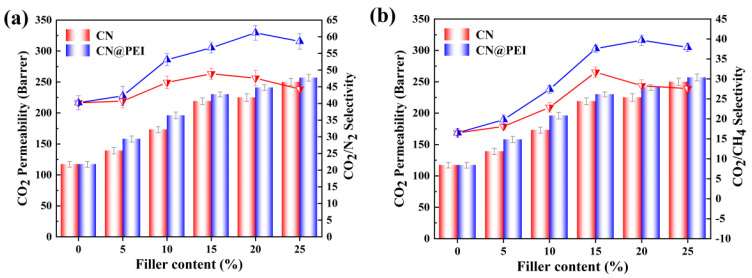
Effect of different filler contents on the gas separation performance of membranes in the dry state: (**a**) CO_2_ permeability and CO_2_/CH_4_ selectivity of membranes; (**b**) CO_2_ permeability and CO_2_/N_2_ selectivity of membranes (membranes tested at 2 bar, 25 °C).

The CO_2_ permeability and selectivity for the prepared MMMs was studied to evaluate their gas separation performance (Figure 12 and Table S2). The introduction of CN and CN@PEI nanosheets significantly affected the Pebax membranes’ permeability. Specifically, the CO_2_ permeation flux of the Pebax/CN membrane increased from 117 Barrer to 250 Barrer as CN loading increased from 5 wt% to 25 wt%, reflecting a 113.7% enhancement. Concurrently, the selectivity initially increased and then decreased. The selectivity of CO_2_/N_2_ and CO_2_/CH_4_ reached their peaks at 48.9 and 31.7 with CN loading of 15 wt%, respectively, representing a 21.6% and 92.1% increase over that of the pure Pebax membrane. However, when CN loading increased to 20 wt%, the selectivity towards CO_2_ decreased for both CO_2_/N_2_ and CO_2_/CH_4_, while the CO_2_ permeate flux remained elevated. Similar trends of significant enhancements in gas permeability and selectivity were observed with increasing CN@PEI loading, and in Pebax/CN@PEI MMMs. The highest selective separation performance of the membranes was achieved at 20 wt% CN loading with values of 61.2 (CO_2_/N_2_) and 39.7 (CO_2_/CH_4_), reflecting increases of 52.2% and 140.6% over those of the pure Pebax membranes, respectively. Notably, the CO_2_ permeability and CO_2_/CH_4_ selectivity of Pebax/CN@PEI membranes surpassed those of the Pebax and Pebax/CN membranes, indicating that the selective transport of grafted PEI with abundant amino groups enhanced the CO_2_ separation performance.

**Figure 13:**
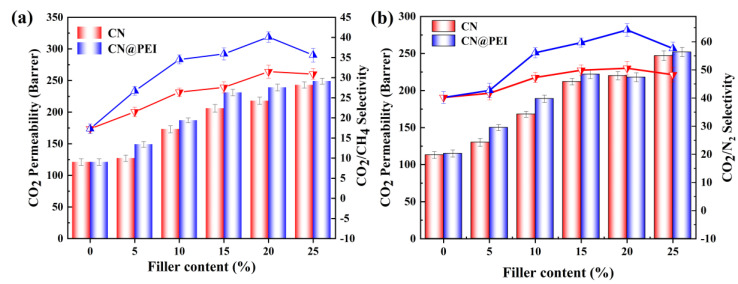
Separation performance of pure membranes and MMMs in (**a**) CO_2_/CH_4_ (50/50 vol%) and (**b**) CO_2_/N_2_ (50 vol%: 50 vol%) gas mixtures.

In order to investigate the membrane separation performance under more practical conditions, mixed gas permeation experiments were conducted on both the pure Pebax membrane and MMMs using CO_2_/CH_4_ (50 vol%: 50 vol%) and CO_2_/N_2_ (50 vol%: 50 vol%) mixed gases at 25 ◦C and 2 bar, and the permeation results are presented in Figure 13 and Tables S3 and S4. As Figure 13 shows, the MMMs exhibited promising mixed gas separation performance, which was generally consistent with the pure gas permeation results. However, differences in the permeation selectivity of the membranes were observed due to the competitive adsorption effect. As the gas mixture passed through the membrane, competitive adsorption and desorption processes occurred between the different gas components (CO_2_/CH_4_ or CO_2_/N_2_) and the membrane material, leading to competition on the membrane surface. When CH_4_ or N_2_ occupied the adsorption sites, it resulted in reduced CO_2_ adsorption. For instance, the 20 wt% MMMs demonstrate a CO_2_ permeability of 239 Barrer with a CO_2_/CH_4_ selectivity of 40.1 during the mixed gas permeation. Such a mixed gas CO_2_ permeability is relatively lower than the pure gas CO_2_ permeability of 241 Barrer, likely owing to the competition effects of CO_2_ and CH_4_ gas molecules in the mixed gas testing. On the other hand, although the mixed gas CO_2_/CH_4_ selectivity of 20 wt% MMMs is slightly lower than their pure gas selectivity, the membranes are still very promising with a CO_2_/CH_4_ selectivity as high as 40.1. These mixed gas permeation results indicate the potential of the membranes for separating gas mixtures under realistic feed conditions.

**Figure 14:**
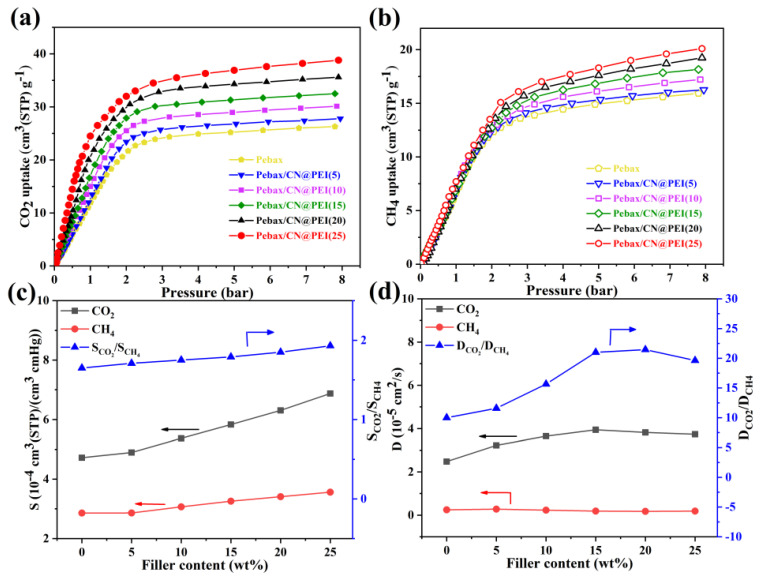
(**a**) and (**b**) gas adsorption isotherms, (**c**) solubility coefficients, and (**d**) diffusion coefficients for pure Pebax membranes and MMMs.

The CO_2_ and CH_4_ adsorption isotherms of the pure Pebax membranes and MMMs were measured to investigate the effect of CN@PEI nanosheets on the gas separation performance (Figure 14a,b). The gas solubility and diffusivity coefficients of pure Pebax membranes and MMMs were calculated (Figure 14c,d). The adsorption of CO_2_ and CH_4_ increased as the CN@PEI nanosheets loading increased (Figure 14a,b), but the CO_2_ adsorption was higher than CH_4_ adsorption, which was attributed to CN@PEI nanosheets’ abundant amino groups, showing more of a distinct affinity towards CO_2_ than CH_4_ molecules. As Figure 14c shows, the solubility coefficients of CO_2_ and CH_4_, as well as the solubility selectivity of CO_2_/CH_4_, significantly increased as CN@PEI nanosheets loading increased compared to the pure Pebax membranes. This observation was consistent with the adsorption isotherm results. However, both CO_2_ diffusivity coefficient and CO_2_/CH_4_ diffusivity selectivity in Pebax/CN@PEI MMMs initially increased then decreased with increasing CN@PEI nanosheets loading (Figure 14d). The uniform dispersion of 2D CN@PEI nanosheets in the membranes provided additional low-resistance transport channels for gas molecules, leading to an increased diffusivity coefficient. Moreover, the diffusion or permeability of gas molecules was greatly influenced by the gas molecules’ kinetic diameter, and CO_2_ molecules with their smaller kinetic diameter compared to CH_4_ typically exhibited higher permeability [44]. The introducing of 2D CN@PEI nanosheets altered the stacking of polymer chains, thereby forming more interfacial barriers that selectively retard gas molecules with larger kinetic diameters (e.g., CH_4_). This consequently enhanced the diffusivity coefficient of CO_2_, which was consistent with the aforementioned DSC results. However, the nanofillers showed pronounced agglomeration tendencies with loadings up to 25 wt%, thereby reducing the effective reaction sites for CO_2_ interaction and decreasing the efficiency of translational transport carriers, ultimately leading to lower diffusivity selectivity.

The incorporation of CN@PEI nanofillers played a crucial role in enhancing CO_2_ capture performance. The underlying causes involved two critical aspects: (1) the abundant amino groups of CN@PEI nanosheets had a higher affinity for CO_2_, thereby improving gas permeance and selectivity; (2) the introduction of 2D CN@PEI nanosheets disrupted the stacking of polymer Pebax chains, creating additional diffusion pathways within the gaps between the nanofillers. Remarkably, agglomeration of the nanosheets occurred as the loading of CN@PEI nanosheets reached 25 wt%, leading to the disorganization of gas transport channels, then gas selectivity decreased.

**Figure 15:**
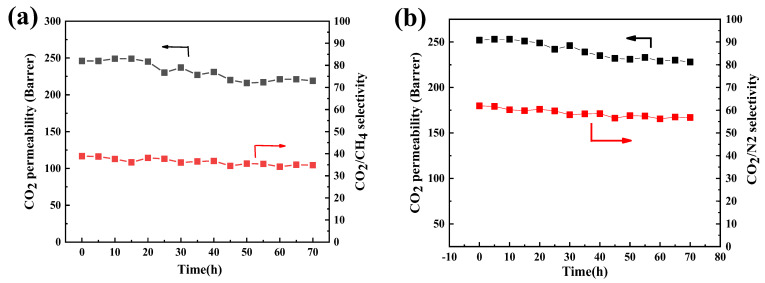
Long-term performance of Pebax/CN@PEI (20) MMMs (membrane tested at 2 bar, 25 °C) in (**a**) CO_2_/CH_4_ (50/50 vol%) and (**b**) CO_2_/N_2_ (50 vol%: 50 vol%) gas mixtures.

Long-term continuous gas permeation tests of Pebax/CN@PEI MMMs with a 20 wt% loading (Pebax/CN@PEI (20) MMM) were conducted to assess the stability of the membrane. As Figure 15a shows, the separation performance of the Pebax/CN@PEI (20) membrane was evaluated in CO_2_/CH_4_ (50/50 vol%) and CO_2_/N_2_ (50 vol%: 50 vol%) mixed gas systems over 70 h. For the full 70 h duration, minor fluctuations in CO_2_ permeability ranging from 216 to 249 Barrer and CO_2_/CH_4_ selectivity from 34.1 to 38.9 Barrer were observed. Furthermore, the long-term stability test results of the mixture gas of CO_2_/N_2_ (50 vol%: 50 vol%) were similar, where CO_2_ permeability ranging from 228 to 252 Barrer and CO_2_/N_2_ selectivity from 56.8 to 61.9 were tested. These results demonstrated that the membranes exhibited exceptional stability during long-term testing, which ascribed to the stabilization of amino groups in the mixed matrix membrane originating from the robust interaction between PEI and CN nanosheets.

### 3.4. Comparison of Gas Separation Performance

**Figure 16:**
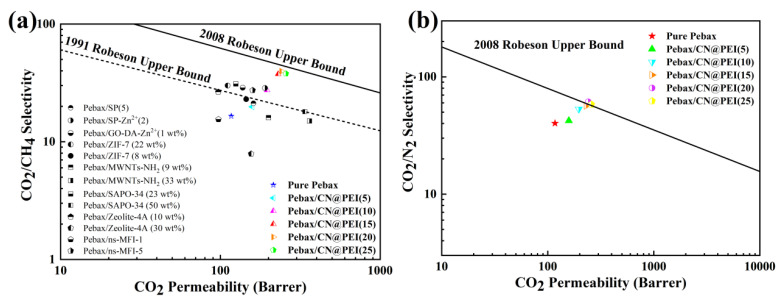
Comparison of CO_2_/CH_4_ separation characteristics of Pebax/CN@PEI MMMs with other reported separation membranes and Robsen upper bound.

Figure 16 and Table S4 demonstrate the separation performance comparison between Pebax/CN@PEI MMMs and Pebax-1657-based MMMs in the reported literatures [45,46,47,48,49,50,51,52]. The results showed that the CO_2_ permeability of Pebax/CN@PEI (25) MMMs was 257 Barrer, which was significantly improved compared to other reported studies. In contrast, the CO_2_/CH_4_ selectivity was only 37.9, which was still lower than the Robeson upper bound and needed to be further improved. It is worth noting that the CO_2_/CH_4_ selectivity of 39.7 for the Pebax/CN@PEI (20) membrane, which was much closer to the 2008 Robeson upper bounds but exceeded the Robeson upper bound, was established in 1991 [46]. The addition of CN@PEI improved the CO_2_ separation performance of the membranes, which was mainly attributed to the CN@PEI nanoparticles with outstanding interfacial compatibility, porosity, and a facilitated CO_2_ transport pathway constructed by amino groups on PEI chains. Moreover, the low-cost raw materials and comparatively straightforward fabrication process enable scalable production while maintaining high performance. This cost-effective approach, combined with the demonstrated separation efficiency, positions g-C_3_N_4_-based hybrid membranes as a more competitive and sustainable alternative to conventional Pebax membranes for industrial gas separation applications. Furthermore, the functionalization of g-C_3_N_4_ nanoparticles with long-chain amine polymers represents a promising approach to enhance the interfacial compatibility of composite membranes, thereby improving their overall separation performance.

## 4. Conclusions

In summary, a novel hybrid membrane was fabricated by embedding PEI-grafted g-C3N4 (CN@PEI) nanosheets into the Pebax matrix. Surface functionalization of g-C3N4 and enhanced polymer–filler compatibility synergistically improved the gas separation performance of MMMs. Compared with unmodified g-C3N4, CN@PEI demonstrated superior compatibility with the Pebax matrix, which effectively mitigated interfacial voids and defects. The incorporation of CN@PEI nanofillers played a crucial role in enhancing CO_2_ capture performance: (1) indicated by CO_2_ and CH_4_ adsorption isotherms, the abundant amino groups of CN@PEI nanosheets had higher affinity for CO_2_, thereby improving gas permeance and selectivity, and (2) the results of solubility coefficients and diffusion coefficients for membranes demonstrated that the introduction of 2D CN@PEI nanosheets disrupted the stacking of polymer Pebax chains, providing additional diffusion pathways within the gaps between the nanofillers. Compared to g-C_3_N_4_, MMMs with CN@PEI loading of 20 wt% exhibited maximum CO_2_ permeability of 241 Barrer as well as CO_2_/CH_4_ and CO_2_/N_2_ selectivity of 39.7 and 61.2, respectively, at 25 °C and 2 bar, approaching the 2008 Robeson upper bound and exceeding 1991 Robeson upper bound. Furthermore, the mixed matrix membrane demonstrated excellent long-term stability, maintaining consistent separation performance over 70 h of continuous operation, which underscored its potential for scalable deployment in carbon capture systems—a critical technology for achieving net-zero emissions.

## Figures and Tables

**Table 1:** Mechanical properties of pure Pebax films and Pebax/CN@PEI MMMs.

Membranes	Breaking Elongation (%)	Young’s Module (MPa)	Tensile Strength (MPa)
Pebax	82.5	11.4	10.9
Pebax/CN@PEI (5)	112.9	12.2	13.5
Pebax/CN@PEI (10)	113.9	12.8	12.2
Pebax/CN@PEI (15)	137.6	16.3	10.4
Pebax/CN@PEI (20)	130.9	15.1	12.6
Pebax/CN@PEI (25)	60.2	11.9	9.8

**Table 2:** Density and FFV of pure Pebax and MMMs.

Membranes	Density (g/cm^3^)	FFV
Pure Pebax	1.042	0.260
Pebax/CN@PEI (5)	1.040	0.265
Pebax/CN@PEI (10)	1.039	0.269
Pebax/CN@PEI (15)	1.037	0.274
Pebax/CN@PEI (20)	1.035	0.278
Pebax/CN@PEI (25)	1.034	0.283

## Data Availability

Data is contained within the article or Supplementary Material. The original contributions presented in this study are included in the article/Supplementary Material. Further inquiries can be directed to the corresponding authors.

## Supplementary Materials

The following supporting information can be downloaded at https://www.mdpi.com/article/10.3390/membranes15100306/s1; Table S1: DSC curves of pure Pebax and MMMs loaded with different nanofillers; Table S2: Effect of filler contents on gas separation performance of membranes at dry state. (membranes were tested at 2 bar, 25 °C); Table S3: Mixed CO2/CH4 (50/50 vol%) separation performance of different membranes; Table S4: Mixed CO2/N2 (50/50 vol%) separation performance of different membranes; Table S5: Comparison of the membrane separation properties in this study with previous Pebax-based membranes reported in the literatures.
