# Supplementary material for: Enhanced CO2 Separation Performance of Mixed Matrix Membranes with Pebax and Amino-Functionalized Carbon Nitride Nanosheets
Source: Membranes (Basel). 2025 Oct 7;15(10):306. doi: 10.3390/membranes15100306 (PMC12566534; doi:10.3390/membranes15100306)
Supplement: Supplementary file 1 [file membranes-15-00306-s001.zip › membranes-3890135-supplementary.pdf]

# Enhanced $CO_2$ separation performance of mixed matrix membranes with amino-functionalized carbon nitride nanosheets

Meng-Ran Hua[a], Qin-Qin Sun[a], Na Li[a,*], Ming-Chao Zhu[a], Yong-Ze Lu[b,c,d,*], Zhao-Xia Hu[a], Shou-Wen Chen[a]

a Jiangsu Key Laboratory of Chemical Pollution Control and Resources Reuse, School of Environmental and Biological Engineering, Nanjing University of Science and Technology, Nanjing 210094, China

b School of Energy and Environment, Southeast University, Nanjing 210096, China

c Key Laboratory of Water Pollution Control and Ecological Restoration of Xizang, National Ethnic Affairs Commission, Xizang Minzu University, Xianyang 712082, China

d Information Engineer College, Xizang Minzu University, Xianyang 712082, China

*Corresponding author:

Na Li: Jiangsu Key Laboratory of Chemical Pollution Control and Resources Reuse, School of Environmental and Biological Engineering, Nanjing University of Science and Technology, Nanjing 210094, China; Email: nli@njust.edu.cn

Yong-Ze Lu: School of Energy and Environment, Southeast University, Nanjing 210096, China; Email: yzlu@seu.edu.cn

**List of Tables:**

**Table S1.** DSC curves of pure Pebax and MMMs loaded with different nanofillers.

**Table S2.** Effect of filler contents on gas separation performance of membranes at dry state. (membranes were tested at 2 bar, 25°C).

**Table S3.** Mixed $CO_2/CH_4$ (50/50 vol%) separation performance of different membranes.

**Table S4.** Comparison of the membrane separation properties in this study with previous Pebax-based membranes reported in the literatures.

**Table S1**. DSC curves of pure Pebax and MMMs loaded with different nanofillers.

| Membranes | $T_g$ (℃) |
|---|---|
| Pebax | -55.01 |
| Pebax/CN@PEI(5) | -53.88 |
| Pebax/CN@PEI(10) | -53.43 |
| Pebax/CN@PEI(15) | -53.25 |
| Pebax/CN@PEI(20) | -53.14 |
| Pebax/CN@PEI(25) | -53.28 |

**Table S2.** Effect of filler contents on gas separation performance of membranes at dry state. (membranes were tested at 2 bar, 25°C).

| Membranes | $CO_2$ Permeance (Barrer) | $CO_2/N_2$ Selectivity | $CO_2/CH_4$ Selectivity |
|---|---|---|---|
| Pebax | 117±4.6 | 40.2±2.1 | 16.5±1.1 |
| Pebax/CN(5) | 139±5.1 | 40.7±2 | 18.1±0.7 |
| Pebax/CN(10) | 173±4.9 | 46.3±1.9 | 22.9±1.2 |
| Pebax/CN(15) | 219±5.5 | 48.9±1.7 | 31.7±1.3 |
| Pebax/CN(20) | 225±6.2 | 47.6±2.3 | 28.3±1.5 |
| Pebax/CN(25) | 250±5.8 | 44.3±2.5 | 27.6±1.2 |
| Pebax/CN@PEI(5) | 158±4.9 | 42.3±2.9 | 19.8±1.2 |
| Pebax/CN@PEI(10) | 196±5.4 | 53.1±1.8 | 27.4±1 |
| Pebax/CN@PEI(15) | 230±3.8 | 56.7±1.6 | 37.6±0.9 |
| Pebax/CN@PEI(20) | 241±4.7 | 61.2±2.2 | 39.7±1.3 |
| Pebax/CN@PEI(25) | 257±5.4 | 58.6±2.3 | 37.9±1.1 |

**Table S3.** Mixed $CO_2/CH_4$ (50/50 vol%) separation performance of different membranes.

| Membranes | $CO_2$ Permeance (Barrer) | $CO_2/CH_4$ Selectivity |
|---|---|---|
| Pebax | 121±5.2 | 17.3±1.2 |
| Pebax/CN(5) | 127±5 | 21.5±1.2 |
| Pebax/CN(10) | 173±5.6 | 26.4±1 |
| Pebax/CN(15) | 206±6.1 | 27.6±1.4 |
| Pebax/CN(20) | 218±5.8 | 31.5±1.6 |
| Pebax/CN(25) | 243±4.9 | 30.9±1.3 |
| Pebax/CN@PEI(5) | 149±4.5 | 26.7±1.1 |
| Pebax/CN@PEI(10) | 187±3.8 | 34.5±1.1 |
| Pebax/CN@PEI(15) | 231±5.1 | 35.9±1.5 |
| Pebax/CN@PEI(20) | 239±5.4 | 40.1±1.3 |
| Pebax/CN@PEI(25) | 249±4.7 | 35.6±1.7 |

**Table S4.** Mixed $CO_2/N_2$ (50/50 vol%) separation performance of different membranes.

| Membranes | $CO_2$ Permeance (Barrer) | $CO_2/N_2$ Selectivity |
|---|---|---|
| Pebax | 113±4.5 | 40.2±2.1 |
| Pebax/CN(5) | 130±5.2 | 41.7±2.4 |
| Pebax/CN(10) | 168±4 | 47.3±1.8 |
| Pebax/CN(15) | 212±4.3 | 49.9±1.9 |
| Pebax/CN(20) | 220±5.5 | 50.6±2.4 |
| Pebax/CN(25) | 247±6.4 | 48.3±2.6 |
| Pebax/CN@PEI(5) | 150±3.9 | 42.7±2.5 |
| Pebax/CN@PEI(10) | 189±4.9 | 56.1±1.8 |
| Pebax/CN@PEI(15) | 222±5.8 | 59.7±1.6 |
| Pebax/CN@PEI(20) | 218±5.9 | 64.2±2.2 |
| Pebax/CN@PEI(25) | 252±6.2 | 57.6±2.3 |

**Table S5.** Comparison of the membrane separation properties in this study with previous Pebax-based membranes reported in the literatures.

| | Membranes | $CO_2$ permeability (Barrer) | $CO_2/CH_4$ selectivity | Testing gas | Temperature (°C) | Pressure (bar) | Ref. |
|---|---|---|---|---|---|---|---|
| 1 | Pure Pebax | 117 | 16.5 | $CO_2/CH_4$ | 25 | 2 | This study |
| 2 | Pebax/CN@PEI (20) | 225 | 28.3 | (50/50 vol%) | | | This study |
| 3 | Pebax/CN (20) | 383 | 8.5 | | | | This study |
| 4 | Pebax/SP (5) | 161 | 21.2 | $CO_2/CH_4$ | 30 | 2 | [45] |
| | Pebax/SP-$Zn^{2+}$(2) | 190.3 | 28.6 | (30/70 vol%) | | | |
| 5 | Pebax/GO-DA-$Zn^{2+}$(1 wt%) | 137.9 | 28.8 | $CO_2/CH_4$ (30/70 vol%) | 30 | 2 | [46] |
| 6 | Pebax/ZIF-7 (22 wt%) | 111 | 30 | Pure gas | 25 | 3.75 | [47] |
| | Pebax/ZIF-7 (8 wt%) | 145 | 23 | | | | |
| 7 | Pebax/MWNTs-$NH_2$ (9 wt%) | 200 | 16 | Pure gas | 35 | 7 | [48] |

| | | | | | | | |
|---|---|---|---|---|---|---|---|
| | Pebax/MWNTs-$NH_2$ (33 wt%) | 361 | 15 | | | | |
| 8 | Pebax/SAPO-34 (23 wt%) | 125 | 21 | Pure gas | 35 | 7 | [49] |
| | Pebax/SAPO-34 (50 wt%) | 338 | 18 | | | | |
| 9 | Pebax/Zeolite-4A (10 wt%) | 97 | 26.5 | Pure gas | 35 | 3.75 | [50] |
| | Pebax/Zeolite-4A (30 wt%) | 155.8 | 7.9 | | | | |
| 10 | Pebax/ns-MFI-1 | 97.3 | 15.5 | Pure gas | 25 | 2 | [51] |
| | Pebax/ns-MFI-5 | 159.1 | 27.4 | | | | |
